# EVALUATION OF ARTHROSCOPIC TREATMENT OF TRIANGULAR FIBROCARTILAGE

**DOI:** 10.1590/1413-785220253305e290745

**Published:** 2025-09-22

**Authors:** Letícia Saka Holanda, Gabriela Júlio Fernandes Viana, Felipe Mariz Queiroz, João Vitor da Cruz Garcia, Fábio Sano Imoto, Eiffel Tsuyoshi Dobashi

**Affiliations:** 1 IFOR Hospital, Rede D’Or Sao Luiz, Sao Bernardo do Campo, Sao Paulo, SP, Brazil.; 2 Universidade Federal de Sao Paulo (UNIFESP), Escola Paulista de Medicina, Sao Paulo, SP, Brazil.

**Keywords:** Magnetic Resonance Imaging, Arthroscopy, Diagnostic Imaging, Wrist Injuries, Triangular Fibrocartilage, Wrist, Ressonância Magnética, Artroscopia, Diagnóstico por Imagem, Fibrocartilagem Triangular, Punho

## Abstract

**Objective:**

To evaluate the clinical outcomes of surgical treatment by wrist triangular fibrocartilage complex (TFCC) lesions videoarthroscopy (VA) and analyze the correlation between magnetic resonance imaging (MRI) and intraoperative VA findings.

**Methods:**

Cross-sectional study of 28 patients undergoing VA from February 2021 to September 2023, selected by predefined inclusion/exclusion criteria. The mean age was 39.6 years (range: 20–58), with a mean follow-up of 13.6 months (6–20). Twelve surgeries (42.9%) were on the right wrist and sixteen (57.1%) on the left. Functional assessment used the 11-item Quick-Dash questionnaire.

**Results:**

Quick-Dash scores were excellent in 18 cases (64.3%), good in 7 (25.0%), and satisfactory in 3 (10.7%). MRI identified 4 (14.3%) extensive and 24 (85.7%) partial tears, while VA confirmed 2 (7.1%) extensive and 26 (92.9%) partial tears. Concordance between MRI and VA was found in 26 cases (92.9%).

**Conclusion:**

Surgical treatment by VA resulted in predominantly excellent functional outcomes. There was high agreement between MRI and VA findings, indicating good sensitivity and specificity. ***Level of Evidence IV; Case Series.***

## RESUMO

**Objetivos:**

Avaliar os resultados clínicos do tratamento cirúrgico por videoartroscopia (VA) das lesões da fibrocartilagem triangular do punho e correlacionar os achados da ressonância magnética (RM) com os observados na VA.

**Métodos:**

Estudo transversal com 28 pacientes submetidos à VA entre fevereiro de 2021 e setembro de 2023, selecionados por critérios de inclusão e não inclusão. A média de idade foi de 39,6 anos (20-58) e o seguimento médio de 13,6 meses (6-20). Do total, 12 cirurgias (42,9%) foram no punho direito e 16 (57,1%) no esquerdo. Para avaliação funcional, utilizou-se o Quick-Dash (11 questões).

**Resultados:**

Na escala Quick-Dash, 18 (64,3%) apresentaram resultados excelentes, 7 (25,0%) bons e 3 (10,7%) satisfatórios. A RM detectou 4 (14,3%) lesões extensas e 24 (85,7%) parciais, enquanto a VA confirmou 2 (7,1%) extensas e 26 (92,9%) parciais. Houve concordância entre RM e VA em 26 casos (92,9%).

**Conclusão:**

O tratamento por VA apresentou resultados clínicos satisfatórios, com maioria de casos classificados como excelentes na avaliação funcional. Verificou-se alta concordância entre RM e VA, demonstrando boa sensibilidade e especificidade dos exames. ***Nível de Evidência IV; Série de Casos.***

## INTRODUCTION

Triangular fibrocartilage complex (TFCC) injuries are common in the genesis of ulnar-side wrist pain, often combined with distal radioulnar joint (DRUJ) instability.

The etiology of pain in the TFCC may be related to both wrist trauma and degenerative processes associated with local wear and tear. In general, this process begins during the third decade of life and progresses in frequency and severity in subsequent decades.

The TFCC is located between the radius, ulna, and semilunar and pyramidal bones of the carpus. This anatomical structure consists of the central disc, the dorsal and volar radioulnar ligaments, the ulnar extensor sheath of the carpus, and the homologous meniscus. This complex has crucial functions in wrist biomechanics, such as stabilizing the ulnocarpal and distal radioulnar joints, distributing load between the ulna and carpus, and facilitating rotational movements of the wrist and forearm.^1,2^

No imaging modality demonstrates perfect sensitivity and specificity for detecting lesions. However, because it is noninvasive, MRI is the most frequently used resource. It offers up to 95% accuracy in detecting TFCC breaks. This technique can replace wrist arthrography; however, in lower-power magnetic fields, MRI has proven to be less accurate, especially for diagnosing peripheral tears, with a sensitivity of 17%.

However, some authors consider wrist AV to be the gold standard for diagnosing TFCC injuries to assess intrinsic wrist ligament injuries, as it allows direct visualization of the wrist joint. Although considered an invasive method, diagnostic arthroscopy is indicated for patients with unequivocal or negative imaging tests, but with a history, symptoms, and clinical examination consistent with the disease.

Considering the different treatment methods, initially conservative treatment with immobilization, modification of activities, corticosteroid injections, and the use of painkillers for a few weeks should be considered first. When symptoms do not go into remission, surgical procedures are recommended. Currently, there is no standard gold standard procedure, and no surgical procedure has been found to be superior.

PRP injection is a recent and commonly used option in the treatment of acute and chronic tendinopathies because it promotes cell viability and collagen synthesis by providing the growth factors TGF-β1 and PDGF-AB.

The *outside-in*, *inside-out, or all-inside* arthroscopic methods allow for appropriate treatment when indicated, where repair with an anchor to perform transosseous suturing is the most commonly used procedure. This feature has the advantage of avoiding open capsulotomy with capsular flap of the original method. This instrument minimizes scarring, soft tissue fibrosis, and joint stiffness. Considering the evaluation of the results, good and excellent results are generally obtained in 60% to 90% of cases.

To evaluate the results, we found that the Visual Analog Scale (VAS), modified Mayo score, arm, shoulder, and hand disability scores, grip strength, and range of motion (ROM) are the primary assessment tools used in the treatment of patients with TFCC injuries.

Therefore, this study aims to evaluate the clinical outcome of arthroscopic surgical treatment of conditions affecting the triangular fibrocartilage of the wrist. As a secondary objective, the authors will evaluate the correlation between the findings of the MRI scans and those found during wrist VA.

## MATERIALS AND METHODS

Initially, this research project was submitted to bioethical evaluation by the Research Ethics Committee of Plataforma Brasil and approved for implementation by CAEE opinion 81673924.2.0000.0087. This is a retrospective cross-sectional cohort study whose sample included patients with TFCC lesions treated surgically between February 2021 and September 2023.

The following criteria were established for inclusion of patients in this study.

1. Patients of both sexes.
2. Aged between 18 and 60 years old.
3. Minimum follow-up period of 6 months.
4. With a diagnosis of traumatic (partial or extensive) or degenerative injury.
5. No previous trauma such as styloid process fractures of the ulna, distal radius fractures, or ARUD injury.
6. No previous history of inflammatory disease
7. Patients with failure of conservative treatment.
8. Patients who accept the terms of the TCLA
9. Patients undergoing MRI and wrist AV for repair or reconstruction of the TFCC.

Those who refused to sign the Informed Consent Form or who had other types of wrist injuries, such as distal radius fractures or previous surgery on the limb under study, were excluded.

Therefore, our case series consisted of 28 patients, 21 (75.00%) men and 7 (25.00%) women, with a mean age at the time of surgery of 39.60 years (minimum of 20 years and maximum of 58 years) and a mean follow-up time of 13.6 months (minimum of 6 months and maximum of 20 months). Twelve (42.86%) were performed on the right wrist and 16 (57.14%) on the left wrist.

Arthroscopic wrist surgeries at the research center in question are performed under general anesthesia with a tourniquet on the upper limb being operated on, with a pressure of approximately 250 mmHg. After sterile preparation, the patient's hand is placed in a traction device and a force of approximately 6 to 7 kg is applied. Using the dorsal approach, portals 3–4 and 4–5 are used for arthroscopy and *shaving*. A *shaver* is used to release the TFCC from the capsule in the *foveal* region. Peripheral and deep tears are visualized through portals 3–4 after release of the TFCC, and instability of this structure is assessed with the aid of a hook. The drill guide is passed through the ulnar surface toward the fovea of the ulna, then a guide wire is passed through and a 2.5 mm suture anchor is inserted. The grip is then released and the repair between the TFCC and the fovea is secured with three knots. The knots are positioned inside the sheath of the ulnar extensor tendon of the carpus to prevent skin irritation. When ligament reconstruction is not necessary, only debridement is performed. After removal of the arthroscope, the portals are sutured with 5-0 nylon. Finally, a sterile dressing is applied and the pneumatic tourniquet is deflated. During outpatient follow-up, the stitches are removed after two weeks and the axillary and palmar immobilization is removed after two weeks when ligament reconstruction is not necessary, or after three weeks when it is necessary. After this period, physical therapy treatment begins.

The Quick-Dash functional assessment method consisting of 11 questions was used. With 11 questions, the tool measures the impact of injuries in these regions, assessing functional limitations, pain, and the ability to perform daily activities.^3^ Its main function is to monitor the progress of treatment and compare the effectiveness of interventions, and it already has a validated Brazilian version.^4^ Responses to QuickDASH were presented based on the relative frequency of responses. The number of injuries identified, as well as their origins and characteristics, in addition to the need for sutures, were presented according to their absolute and relative frequencies in relation to the total sample size.

## RESULTS

In the collective evaluation of responses to Quick-Dash, when patients were asked if they could open a new jar or one with a very tight lid, 76.7% responded that they had no difficulty, while 23.3% responded that they had little difficulty. Regarding the ability to perform heavy household tasks, such as washing walls or floors, 63.3% reported no difficulties, 26.7% reported little difficulty, while 10.0% reported moderate or severe difficulty. Regarding the ability to carry a bag or briefcase, 93.3% reported no difficulties, while 6.7% reported little or moderate difficulty. When asked about their ability to wash their own backs, 86.7% reported no difficulty, with 13.3% reporting minor difficulty. When asked about their ability to use a knife to cut food, 93.3% of patients reported no difficulty, with 6.7% reporting minor difficulty. Regarding the ability to participate in recreational activities that require some strength or impact on the arms, shoulders, or hands, such as playing volleyball or hammering, 66.7% of participants reported no difficulties, 23.3% reported little difficulty, and 10% reported moderate difficulty. In the assessment that identified the extent to which the wrist problem affected normal activities with family, friends, neighbors, or colleagues, 80% of patients responded that activities were not affected, 16.7% responded that activities were slightly affected, while 3.3% stated that such activities were moderately affected. When asked whether the wrist problem affected their work or even limited their regular daily activities in the last week, 76.7% said there were no limitations, while 23.3% reported few limitations. Pain in the arm, shoulder, or hand was not reported by 73.3% of patients, while mild pain was reported by 26.7%. Skin discomfort (pinching) in the arm, shoulder, or hand was not reported by 90% of patients, being present in 10% of the sample (mild discomfort). Finally, when patients were asked whether they had difficulty sleeping because of pain in their arm, shoulder, or hand, 90% reported no difficulties, while 10% reported minor difficulties.

Soon after administering the Quick-Dash questionnaire, we obtained 18 (64.28%) excellent, 7 (25.00%) good, and 3 (10.72%) satisfactory results. MRI identified 4 (14.29%) extensive lesions and 24 (85.71%) partial lesions, while VA identified 2 (7.14%) extensive lesions and 26 (92.86%) partial lesions. With regard to agreement between RM and VA, we observed 26 (92.86%) cases.

## DISCUSSION

Suspected TFCC damage is diagnosed through appropriate physical examination, which allows for a reliable diagnosis of foveal tears. Palpation of the bone near the ulnar styloid often causes pain, and when combined with a positive stress test, such as foveal compression, indicates a rupture. Occasional additional clicks may also indicate the presence of ARUD instability.

However, it is indisputable that diagnosis depends on resources such as radiography, arthrography, MRI, magnetic resonance arthrography (MRA), and wrist VA. However, there is no consensus regarding the sensitivity, specificity, and accuracy of such diagnostic imaging resources, despite their frequent use.

The diagnostic values of this modality range from 71 to 100% for central and radial ruptures of the TFCC. However, the diagnostic performance of conventional MRI for detecting peripheral tears is only 17%. This percentage is justified by the presence of high-sign vascularized fibrous tissue between the two ulnar insertions mimicking the rupture.

According to some studies, MRI is up to 95% accurate in detecting foveal tears of the TFCC complex.^5^ This technique is sensitive for diagnosing such lesions, but according to some authors, arthroscopy appears to be the most accurate method for diagnosis.

However, according to hand orthopedic surgeons, the gold standard for evaluating intrinsic wrist ligament injuries is arthroscopy. His argument relates to the possibility of directly visualizing the articulation of the wrist. However, we also found divergent opinions questioning the degree of diagnostic confidence. It is considered an invasive and expensive method, so VA would be restricted to patients with unequivocal or negative imaging studies despite having a history, symptoms, and clinical examination consistent with the disease.

For ARM, we found the study by Haims et al.^6^, which evaluated 86 MRI exams and found sensitivity of 17%, specificity of 79%, and accuracy of 64%. In the study by Morley et al.^7^, we found 54 MRI studies that demonstrated sensitivity of only 44% and specificity of 87%.

The diagnostic accuracy of MRI increases with magnetic field strength (3 Tesla), coil technology, and pulse sequencing compared to examinations on 1.5 T machines.

In a study conducted by Smith et al.^5^ in a systematic review that included 21 studies (982 wrists), we found that the sensitivity of MRI in detecting full-thickness ruptures of the LCC was 75% and the specificity was 81% with stronger fields. Your study recommended that ARM should be preferred over conventional MRI. The findings of Spies et al.^8^ studies corroborate the outcomes described above. However, we should note that ARM is an invasive procedure that can cause pain and discomfort in the wrist due to the administration of intra-articular contrast. Due to synovial irritation. In rare cases, serious complications such as joint infection should be remembered. We believe that the absence of a standardized protocol for performing MRI in the evaluation of TFCC may contribute to low diagnostic accuracy. Variations in MRI parameters, such as magnetic field strength and contrast agent use, are not effective in detecting TFCC lesions. On the other hand, VA performed with specific portals allows a comprehensive assessment of the wrist structures, facilitating accurate classification of injuries according to Palmer's classification.^9^ In this case, standardization of MRI could contribute to improving diagnostic accuracy and allow replication of results in different centers.

The importance of wrist AV in the diagnosis and treatment of TFCC injuries is indisputable. We highlight its importance in allowing arthroscopic inspection to achieve a correct diagnosis of injuries, as well as their morphological characteristics such as location, shape, and dimensions. Such assessments are possible thanks to the magnified view of the structures studied and the possibility of using various portals. They also allow visualization of the radiocarpal and midcarpal joints to diagnose and treat coexisting injuries.

Furthermore, arthroscopic techniques theoretically offer advantages, including smaller incisions, less tissue dissection, faster recovery, and greater patient satisfaction.

Our study compared the diagnostic efficacy of MRI in relation to VA. We observed that MRI detected TFCCP lesions in 100% of cases, with 4 (14.28%) being extensive and 24 (85.71%) partial. The VA showed 2 (7.14%) extensive lesions and 26 (92.85%) extensive lesions. However, the degree of agreement between the tests used in our study was 92.85%, with two cases showing no agreement regarding the extent of the damage. We agree with the opinion of several authors that there are advantages to VA. Still, the degree of agreement between the resources evaluated was considered excellent according to the Kappa classification (with the highest number of categories). The population sample evaluated in our Service, although small, indicates that arthroscopy allows direct and detailed visualization of intra-articular structures, enabling immediate interventions, as recommended in the literature.

Fleiss's Kappa is derived from Cohen's Kappa. The main difference between Fleiss's Kappa statistic and Cohen's Kappa is that Fleiss's Kappa allows us to assess the degree of agreement between three or more observers/evaluators. In contrast, Cohen's Kappa allows us to analyze only two. The value of the Kappa agreement coefficient can range from (-*p_e_*/1-*p_e_*) to 1. The closer the value is to 1, the greater the indication that there is agreement among the evaluators, and the closer it is to zero, the greater the indication that the agreement is purely random.

Based on the results observed, arthroscopy showed the same sensitivity of 100% in identifying lesions requiring surgical intervention. However, diagnostic accuracy in indicating the best treatment depended on the information obtained from its use. This suggests that, although MRI is highly specific, its sensitivity may be compromised, indicating that the severity of some lesions may not be accurately diagnosed by this method. The results of this study corroborate the findings of previous studies, showing that the diagnostic accuracy of MRI is inferior to arthroscopy, even considering technical variations among radiologists.^10^

We looked at the functional results across the board after the Quick-Dash questionnaire was completed and found that

Finally, it should be noted that the surgical procedures performed in our Service were effective, as no significant changes were noted in the Quick-Dash scale scores. These data confirm the findings of Yao and Lee,^11^ who reported excellent results in patients undergoing arthroscopy for the correction of wrist injuries.

The limitations of this study are related to several factors. Its retrospective design with inherent biases, small sample size, and lack of some clinical elements may interfere with the results. However, there is complete documentation with good quality images obtained. We believe that the evaluation of MR and ARM results, as well as the results of diagnostic arthroscopy, depend on the personal experience of the radiologists who perform the diagnostic interpretations, and the results are directly related to the expertise of the orthopedic surgeon who performs them.

We may not have documentation of low-grade complications such as skin problems or postoperative pain, as we did not find any such records. Other potential complications, though subtle and transient, could include sensory nerve damage. This study comprised a series of cases in which there was no randomization.

## CONCLUSIONS

The study highlights the high specificity and sensitivity of MRI and VA for diagnosing TFCC lesions that require surgical intervention. The combination of advanced imaging technologies and minimally invasive techniques, such as arthroscopy, can significantly improve clinical outcomes by promoting more accurate diagnoses and effective treatments.

When the Quick-Dash questionnaire was applied, we observed that 18 (64.28%) patients obtained results classified as excellent, 7 (25.00%) as good, and 3 (10.72%) as satisfactory. The study revealed high specificity and sensitivity between MRI and VA examinations, with agreement in 26 (92.86%) cases.
